# First Genome Sequence of Newcastle Disease Virus of Genotype VIIi from Jordan

**DOI:** 10.1128/MRA.01136-18

**Published:** 2018-12-13

**Authors:** Mustafa Ababneh, Helena L. Ferreira, Mohammad Khalifeh, David L. Suarez, Claudio L. Afonso

**Affiliations:** a Department of Basic Medical Veterinary Sciences, Jordan University of Science & Technology, Irbid, Jordan; b U.S. National Poultry Research Center, Southeast Poultry Research Laboratory, Athens, Georgia, USA; c Department of Veterinary Medicine, Faculty of Animal Science and Food Engineering, University of São Paulo (FZEA-USP), Pirassununga, São Paulo, Brazil; KU Leuven

## Abstract

Newcastle disease virus (NDV) was detected by reverse transcriptase PCR (RT-PCR) from total RNA isolated from a chicken spleen of a backyard flock in Jordan. The complete coding genome sequence of NDV/chicken/Jordan/J11-spleen/2018 was obtained with MiSeq (Illumina) sequencing. Phylogenetic analysis of the concatenated coding sequences classified the virus as class II subgenotype VIIi.

## Abstract (precis)

Newcastle disease virus (NDV) was detected by reverse transcriptase PCR (RT-PCR) from total RNA isolated from a chicken spleen of a backyard flock in Jordan. The complete coding genome sequence of NDV/chicken/Jordan/J11-spleen/2018 was obtained with MiSeq (Illumina) sequencing.

## ANNOUNCEMENT

Newcastle disease virus (NDV), family *Paramyxoviridae*, genus Avulavirus (1), causes a deadly disease of poultry that remains an ongoing threat in countries of endemicity (2). A 4-month-old chicken flock with 150 birds in Irbid City in the north of Jordan had 80% mortality within 2 weeks in January 2018. Diarrhea and neurologic clinical signs consistent with Newcastle disease were observed in some birds before the mortality. Sick birds were necropsied, and gross lesions and hemorrhage in the proventriculus were seen. Proventriculus, spleen, intestine, liver, heart, and lung samples were collected for laboratory testing and determined to be NDV positive with reverse transcriptase PCR (RT-PCR).

**FIG 1:**
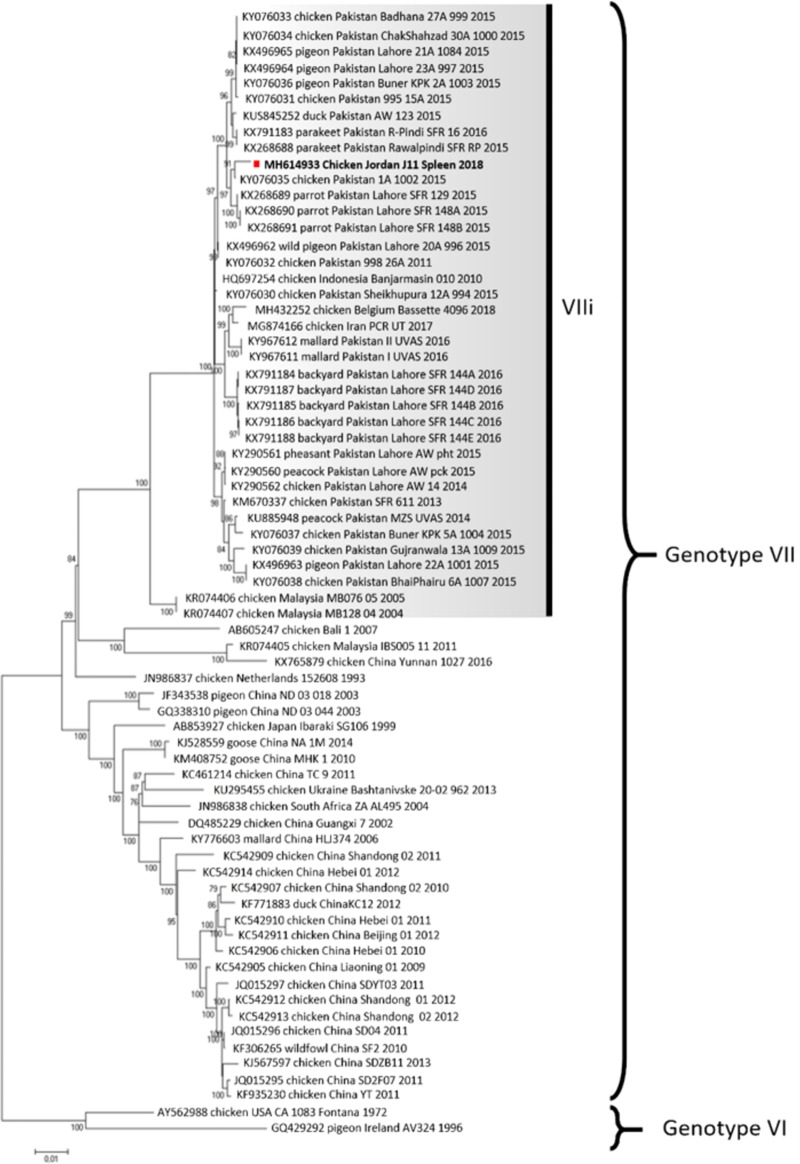
Evolutionary history was inferred by using the maximum likelihood method based on the general time-reversible model. The tree with the highest log likelihood (−61,001.55) is shown. Percentage of trees in which the associated taxa clustered together is shown next to the branches. The initial tree(s) for the heuristic search was obtained automatically by applying Neighbor-Join (NJ) and BioNJ algorithms to a matrix of pairwise distances estimated using the maximum composite likelihood (MCL) approach and then selecting the topology with the superior log-likelihood value. A discrete gamma distribution was used to model evolutionary rate differences among sites (5 categories [+G, parameter = 0.3664]). The tree is drawn to scale, with branch lengths measured in the number of substitutions per site. Analysis involved 70 nucleotide sequences. Codon positions included were first, second, third, and noncoding. All positions containing gaps and missing data were eliminated. A total of 13,744 positions were in the final data set of concatenated coding sequences of 6 NDV genes. The sample NDV/chicken/Jordan/J11-spleen/2018 sequence is highlighted with a red square.

RNA extraction from fresh spleen tissue was performed with TRIzol (Ambion-Thermo Fisher Scientific) followed by purification with a Direct-zol kit (Zymo Research). RNA was quantified with spectrophotometry and Qubit (Invitrogen) fluorimetry. RNA was reverse transcribed, and DNA libraries for next-generation sequencing (NGS) were prepared using the KAPA stranded RNA-Seq library preparation kit (Kapa Biosystems, USA) according to the manufacturer’s instructions. The size distribution and concentration of DNA in the prepared libraries were checked on a Bioanalyzer 2100 and a Qubit instrument using a high-sensitivity (HS) DNA kit (Agilent Technologies, Germany) and a Qubit double-stranded DNA (dsDNA) HS assay kit (Life Technologies, USA), respectively (3). Paired-end sequencing (2 × 250 bp) of the generated libraries was performed on a MiSeq instrument with the 500-cycle MiSeq reagent kit version 2 (Illumina, USA). Raw sequence data were analyzed and assembled with MIRA version 3.4.1 within a customized workflow on the Galaxy platform as described previously (4). In total, 2,000,891 paired-end reads were generated. The final contig based on an aligment with other NDV strains was 15,159 bp long and missed 21 and 12 nucleotides at the 3′ and 5′ untranscribed regions (UTR), respectively, which corresponds to a genome coverage of 99.8%. This contig was made up of 9,671 reads and had an average depth of coverage of 89.4-fold with a maximum of 209. For phylogenetic analysis, complete NDV genome sequences closely related to the studied viruses were downloaded from GenBank (n = 70). The concatenated coding sequences of six NDV genes were used to construct final phylogenetic trees with MEGA7 (5). The maximum likelihood method based on the general time-reversible (GTR) model (6) with a discrete gamma distribution (5 categories [+G, parameter = 0.3664]) was used (Fig. 1). The current classification criteria (7) for naming subgenotypes and genotypes were followed in this study. Pairwise distances with 1,000 bootstrap replicates showed a high genetic identity (99.3%) to the isolate characterized as genotype VIIi, class II, of Newcastle disease virus in Pakistan (GenBank accession number KY076035) (Fig. 1). This is the first identification of a NDV VIIi strain, named NDV/chicken/Jordan/J11-spleen/2018, in a chicken flock from Jordan.

### Data availability.

The complete genome sequence of Newcastle disease virus genotype VIIi has been deposited in GenBank under accession number MH614933.
